# Oral bacterial flora of Indian cobra (*Naja naja*) and their antibiotic susceptibilities

**DOI:** 10.1016/j.heliyon.2018.e01008

**Published:** 2018-12-17

**Authors:** Sujogya Kumar Panda, Laxmipriya Padhi, Gunanidhi Sahoo

**Affiliations:** a Department of Zoology, North Orissa University, Baripada-757003, Odisha, India; b Department of Zoology, Utkal University, Bhubaneswar-751004, Odisha, India

**Keywords:** Bioinformatics, Microbiology, Veterinary science, Zoology

## Abstract

**Objectives:**

The objective of the present work was to examine the bacterial flora associated with the oral cavity of Indian cobra and to study their antibiogram.

**Methods:**

Oral swabs, collected from six healthy (4 males and 2 females) adult cobra, were subjected to microbiological examination through differential media. A total of 74 isolates which demonstrated noticeable colony characters were studied with different biochemical tests. The strains that showed distinctive colonies, morphology and biochemical parameters were additionally subjected to phylogenetic characterization using 16S rRNA gene sequences. Further, the isolates were subjected to antimicrobial susceptibility testing using ICOSA-20-plus and ICOSA-20-minus.

**Results:**

Microscopic examination of the oral cavity of Indian cobra revealed the dominance of Gram-negative bacteria over Gram-positive. The oral microflora constituted of bacteria such as *Salmonella* sp. (*S. typhi, S. paratyphi* A); *Pseudomonas* sp. (*P. aeruginosa, P. fluorescence*); *Proteus* sp. (*P. mirabilis, P. penneri, P. vulgaris*); *E. coli*; *Morganella* sp.; *Citrobacter* sp. (*C. diversus, C. freundii*); *Aeromonas* sp. (*A. hydrophila, A. salmonicida*)*; Enterobacter* sp. (*E. aerogens*); *Acinetobacter* sp. (*A. baumannii*); *Neisseria* sp.; *Serratia* sp.; *Bacillus* sp. (*B. cereus, B. megatarium, B. atrophaeus* and *B. weihenstephanensis*); *Enterococcus* sp. (*E. faecalis, E. faecium*); *Staphylococcus* sp. (*S. aureus, S. epidermidis*); *Alcaligenes* sp.; *Chryseobacterium* sp. and *Micrococcus* sp. Most of the isolates were resistant towards antibiotics such as Penicillin, Cefpodoxime, Amoxyclav, Co-Trimoxazole, Ticarcillin, Erythromycin and Nalidixic acid while sensitive towards Ciprofloxacin, Gentamicin, Ofloxacin, Sparfloxacin, Tobromycin, Ceftriaxone, Tetracycline, Novobiocin and Imipenem.

**Conclusions:**

The secondary complications of the snake bite victims should be managed with appropriate antibiotics after proper examination of the bacterial flora from the wound sites.

## 1 Introduction

Snakes are distributed throughout the world and considered as threat to public health. Recent surveys reported 1220000–5500000 snakebite cases per annum globally, out of which 125000 cases lead to death or disability. An estimated 4 million cases occur annually in Asia, most being in southeastern parts [1]. India registers about 200000 snakebite cases annually but the fatality rate is not exactly known. The number of deaths varies from 1000 to 50000 as reported by different Government agencies. The variation is due to the fact that most victims of snakebite opt for village-based traditional therapists, not government hospitals. This massive statistical discrepancy has significant and urgent consequences. Mohapatra et al. [2], estimated 123000 snakebite deaths from 6671 randomly selected areas during the period 2001–2003. In India, the annual snakebite deaths were highest in the states of Uttar Pradesh (8700), Andhra Pradesh (5200) and Bihar (4500). Odisha, the eastern coastal state, registers a death rate of 5.6 per 100000 cases. People in rural areas, primarily farmers, laborers and their family members, when affected by snakebites, not always have treatment available.

In the Indian subcontinent, almost all snakebite deaths have traditionally been attributed to the big four snakes, consisting of the Russell's viper, Indian cobra, saw-scaled viper, and the common krait. “*Naja naja*” (Linnaeus, 1758), commonly known as cobra and seen in large numbers in Odisha, is a potentially harmful snake as it inhabits around human habitations, paddy fields, bushy forests both in rural and even urbanized areas [3]. Fifty percent of snakebite deaths in Odisha is due to cobra bite and has later complication like local necrosis and sloughing of skin which takes several months to recover [3]. This extensive necrosis may be due to both venom and the contaminated microflora. Hence, the aim of the present study was to examine the associated bacteria from the oral cavity of healthy Indian cobra and study of their antibiogram.

## 2 Materials and methods

### 2.1 Ethical approval

All experiments have been conducted as per the guidelines of the Institutional Animal Ethical Committee of North Orissa University which follow CPCSEA guidelines. Permission for the work obtained from the Principal Chief Conservator of Forests (Department of Forests and Environment, Government of Odisha).

### 2.2 Collection of snakes

All the snakes used in this study (Table 1) were captured from various localities (household) of Odisha by a snake rescue team (working since 2005 with assistance from the Rufford Foundation and Department of Forests and Environment, Government of Odisha). After capture, the snakes were brought to Department of Zoology, North Orissa University for species identification with a qualified and experienced team. The team has identified over 2000 snake cases since the year 2005 which were later released back into the wild. The snakes were transferred separately in cloth bags and locked within a ventilated box. They were not given any food, drugs or antibiotics. The mouth swabs were taken after 7 day of capture. Physically inactive (unhealthy) snakes and snakes too small to produce a satisfactory oral swab were excluded from the study. The snakes were released back to the wild immediately after processing.

**Table 1:** Data sheet regarding collection of *Naja naja* for oral microflora study.

Scientific name, English name, Local name	Designation of individuals	Date and place of collection	Temperature during collection	Habitat	Gender
*Naja naja* (Linnaeus, 1758), Binocellate cobra, Naga/Gokhar sapa	21	18.02.2010; Kamakhyanagar	15 °C	School	Male
	24	22.02.2010; Bhubaneswar	22 °C	Rice field	Female
	28	24.02.2010; Bhubaneswar	22 °C	Rice field	Female
	40	20.05.2010; Balasore	35 °C	Kitchen	Male
	59	30.05.2010; Baripada	37 °C	House	Male
	60	30.05.2010; Baripada	37 °C	House	Female

### 2.3 Swabbing procedure

The mouth of the snakes were opened by experts with the help of sterile mouth gags to facilitate swabbing of the oral cavity. Two oropharyngeal swab samples were collected from each snake using sterile cotton tipped swab sticks. Swabs were taken by rotating the cotton tip on the floor of the oral cavity and spread immediately on different aerobic culture media like Cetrimide agar (CA), Eosin Methyl Blue agar (EMB), Littman Oxgall agar (LOA), MacConkey (MAC), Nutrient agar (NA), Phenolphthalein Phosphate agar (PPA), Thiosulphate Citrate Bile salts Sucrose agar (TCBS) and Xylose Lysine Deoxycholate agar (XLD). The spread plates were incubated for 24–48 h at 37 °C.

### 2.4 Bacterial identification

The isolated strains were first identified based on their colony morphology and Gram character. Further, the strains were subjected to different biochemical characters viz. Catalase, Oxidase, Motility test, Indole, Methyl red, Voges Proskauer, Citrate, utilization of sugars and production of H_2_S in Triple sugar iron agar slant [4]. Growth of the bacteria were checked at different NaCl concentrations, temperature and pH ranges, fermentation of sugars such as arabinose, mannitol, xylose, glucose, lactose, citrate and utilization of amino acids arginine and lysine decarboxylase test. The production of extracellular enzymes namely caseinase, protease, gelatinase and lipase was studied [4].

### 2.5 16S rRNA gene sequencing of the isolates

Few isolates were subjected to 16S rRNA gene sequencing based on distinctive colonies, morphology and biochemical parameters. The isolates were sub-cultured from −80 °C in 25% glycerol on MHA agar. Phylogenetic characterization of the isolates was carried out using 16S rRNA gene sequences amplified using with three universal primers 5′- AGA GTT TGA TCC TGG CTC AG -3′; 5′- CCC ACT GCT GCC TCC CGT AG -3′; 5′- TAA CAC ATG CAA GTC GAA CG -3′; 5′- GTA TTA CCG CGG CTG CTG -3′; 5′- CTA CGG GAG GCA GCA GTG GG -3′ and 5′- CCG TCA ATT CCT TTG AGT TT -3′. The amplified products were purified, and sequencing was carried out at Macrogen (Seoul, South Korea). The 16S rRNA gene sequence of the isolates were aligned using Maximum Likelihood method in MEGA6 based on the General Time Reversible model, with initial tree obtained by Neighbor-Joining method and evolutionary rate difference among sites was modelled using (=0.2841), along with percentage of sites (34.9593% sites). The tree with the highest log likelihood (−8104.28) is shown and the bootstrap values are shown above the branches. The analysis involved 41 nucleotide sequences. The BLAST program from the NCBI (National Center for Biotechnology Information) database was used to identify the closer related species to the bacterial strain. The gene bank accession number of the strains are KX164444, KX495210, MF084216 and MF084215.

### 2.6 Antibiotic sensitivity assay

The isolates were tested with two groups of antibiotics: ICOSA-20-plus and ICOSA-20-minus (Himedia, India). The inhibition zones were measured with Himedia scale and scored as sensitive, intermediate susceptibility and resistant according to CLSI guidelines [5]. The antibiotic susceptibility test of all isolates was performed with Muller Hinton agar at 37 °C for 24 h.

## 3 Results and discussion

**Fig. 1:**
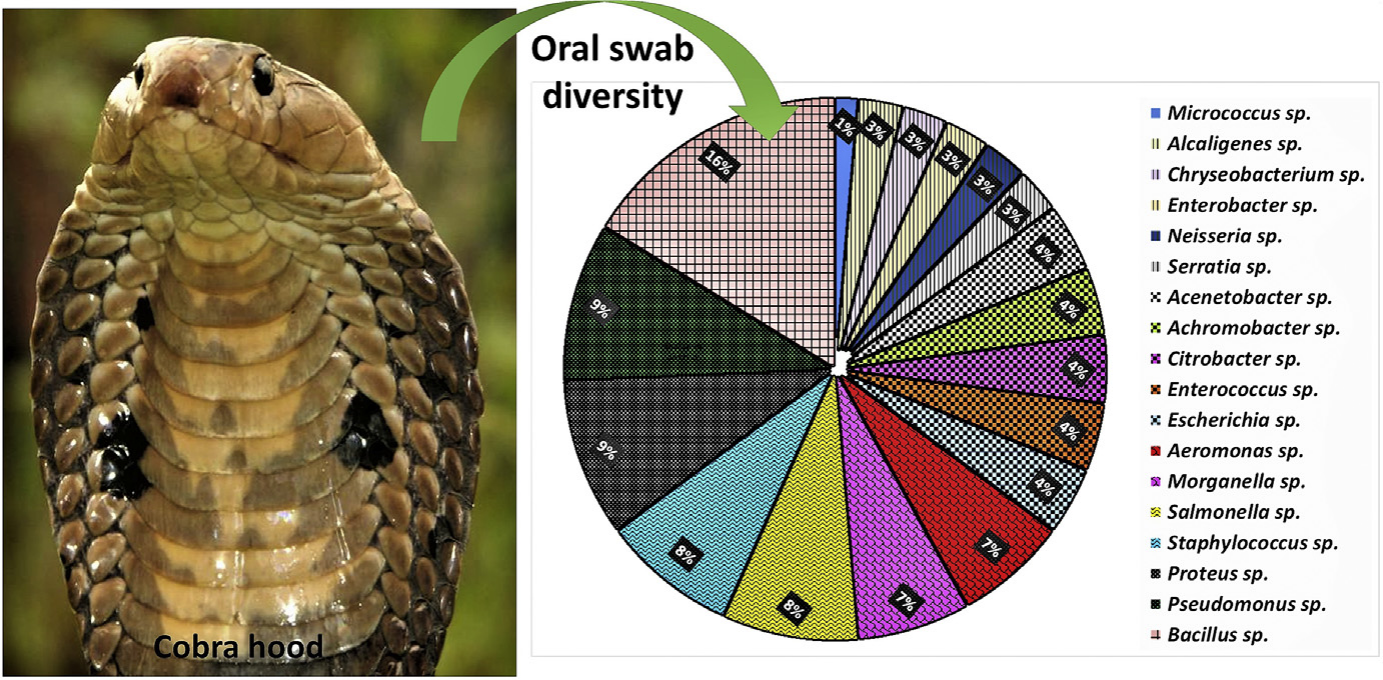
Eighteen genera of bacteria isolated from oral cavity of *Naja naja*.

The bacteria associated with the oral cavity of *N. naja* were successfully isolated and characterized. The mouth cavity was shown to harbor diverse and abundant bacterial communities. A total of ninety-five colonies were isolated out of which seventy-four demonstrated noticeable colony characters and were selected for different biochemical tests. All the isolates were grouped into different Genera and species based on their similarities among biochemical features. Most of the bacteria were Gram-negative, motile with the presence of flagella. A total of 57 isolates were identified to 20 species with 18 genera while 11 isolates remained unidentified (Fig. 1).

Among Gram-negative members, *Pseudomonas* and *Proteus* were the dominant genera followed by *Salmonella, Morganella* and *Aeromonas.* Among others, *E. coli* and *Acinetobacter* species have proportionate distribution. *Alcaligenes, Citrobacter, Enterobacter, Chryseobacterium* and *Serratia* sp. were the minor components (Table 2). Among Gram-positive members, *Bacillus* and *Staphylococcus* dominated over other bacteria such as *Enterococcus* and *Micrococcus* (Table 3). All strains were subjected to different sugar fermentation test to identify species level (Table 4).

**Table 2:** Biochemical characters among Gram-negative rods and bacilli isolated from oral cavity of *Naja naja*.

Test No.	40M1, 60M1	28C1, 59C2	21C1, 24C1, 28C3, 40C1, 59C1, 60C2	21T1, 28T2, 59T2, 60T2	60X3, 21X1	28X1, 24X1, 60X2, 59X1	21X2, 28X2, 40X1, 60X1	28E2, 28M1, 59M3	21E1, 59E2	28N2, 60N1	21C2, 28C2, 59M1	24M2, 28M2, 40M2, 59M2, 60M2
1.	**+**	**+**	**+**	**+**	**+**	**+**	**+**	**+**	**+**	**+**	**+**	**+**
2.	−	**+**	**+**	**+**	−	**+**	−	−	**+**	**+**	−	−
3.	−	−	**+**	−**/+**	−	**+/+**	−	**+**	−	**+**	**+**	**+**
4.	−	−	−	−	−	**+**	**+**	**+**	−	−	−	**+**
5.	**+**	−	−	**+**	−	−	−	−	**+**	**+**	−	−
6.	**+/**−	−**/+**	**+**	−	**+**	**+/**−	−	−	**+**	−	**+**	**+/**−
7.	**+**	**+**	−	**+**	**+**	**+**	**+**	**+**	**+**	**+**	−	−−
8.	**+**	−	−	**+**	−	**+**	−	−	**+**	−	**+**	−
9.	−	−	−	**+**	−	−	−	−	−	−	−	−
10.	**+**	−	−	−	−	−**/+**	−	**+**	−	−	−	−
11.	−	−	−	−	**+**	**+**	**+**	−	−	−	−	−
12.	−	−	−	−	−	−	−**/+**	−	−	**+**	−	−
13.	−	**+**	**+**	**+**	−	−	**+**	−	−	−	−	−
14.	−	−	−	**+**	−	−	−	−	−	**+**	−	−
15.	**+**	−	−	−	−	−	−	−	−	**+**	−	−
16.	**+**	−	−	**+**	−	**+/**−	−	−	−	**+**	−	−
17.	**+**	−	**+**	**+**	−	**+**	−	**+**	−	**+**	−	−
18.	−	−	−	−	−	**+**	−	**+**	**+**	**+**	−	−
19.	−	−	**+**	**+**	**+**	−	−	−	−	**+**	−	−
20.	−	−	**+**	**+**	**+**	**+**	−	−	−	**+**	−	−
	*Serratia* sp.	*Alcaligenes* sp.	*Pseudomonas* sp.	*Aeromonas* sp.	*Citrobacter* sp.	*Proteus* sp.	*Salmonella* sp.	*E. coli*	*Enterobacter* sp.	*Chryseobacterium* sp.	*Acenetobacter* sp.	*Morganella* sp.

1. Catalase; 2. Oxidase; 3. Indole; 4. Methyl red; 5. Voges Praskuer; 6. Citrate; 7. Utilization of glucose; 8. Utilization of sucrose; 9. Utilization of lactose; 10. Production of gas; 11. H_2_S production; 12. Arginine; 13. Lysine; 14. Starch; 15. Esculin; 16. Urea; 17. Gelatin; 18. Nitrate; 19. Growth above 42 °C; 20. Growth at 7% NaCl.

**Table 3:** Biochemical characters among Gram-positive rods, bacilli and coccus isolated from oral cavity of *Naja naja*.

Test	21N1, 24N2, 28N3, 40N1, 59N1, 60N2	24N1, 40E1, 59E3	28N1	21P1, 24P1, 28P2, 40P1, 59P1, 60P2	24E1, 40E2
1.	**+**	−	**+**	**+**	−**/+**
2.	**+**	−	−	−	**+**
3.	**+/**−	−	−	−	−
4.	**+/**−	**+**	−	−	−
5.	**+**	−	**+**	−	**+**
6.	**+**	**+**	−	**+**	−
7.	**+**	−	**+**	**+**	−
8.	**+/**−	−	−	**+**	−
9.	−	−	−	**+**	−
10.	**+/**−	−	−	−	−
11.	−	−	−	−	−
12.	**+/**−	**+**	−	−	−**/+**
13.	**+/**−	−	**+**	−	−
14.	**+**	−	−	−	−
15.	**+/**−	−	−	−	−
16.	**+/**−	−	−	**+**	−
17.	**+**	−	−	−	−
18.	**+**	−	**+**	−	−
19.	**+**	**+**	−	−	−
20.	**+**	**+**	−	−	−
	*Bacillus* sp.	*Enterococcus* sp.	*Micrococcus* sp.	*Staphylococcus* sp.	*Achromobacter* sp.

1. Catalase; 2. Oxidase; 3. Indole; 4. Methyl red; 5. Voges Praskuer; 6. Citrate; 7. Utilization of glucose; 8. Utilization of sucrose; 9. Utilization of lactose; 10. Production of gas; 11. H_2_S production; 12. Arginine; 13. Lysine; 14. Starch; 15. Esculin; 16. Urea; 17. Gelatin; 18. Nitrate; 19. Growth above 42 °C; 20. Growth at 7% NaCl.

**Table 4:** Differential sugar fermentation test for isolates from the oral cavity of *Naja naja*.

Strain	a	b	c	d	e	f	g	h	i	j	k	l	Species identification
21C2, 28C2	+	+	−	−	+	+	−	+	−	−	−	+	*Acenetobacter baumannii*
21T1, 28T2, 40T1, 59T2	+	−	−	+	−	−	+	−	−	+	+	+	*A. Aeromonas hydrophila*
60T2	+	+	+	−	−	−	+	−	−	+	+	+	*A. Aeromanas salmonicida*
59M1	+	−	−	−	−	−	−	+	−	−	−	+	*Acinetobacter* sp.
28C1, 59C2	+	−	−	−/+	−	−	−	−	−	−	−/+	−	*Alcaligenes* sp.
28N3, 59N1	+	−	−	−	−	−	+	−	−	−	+	−	*Bacillus cereus*
24N2	+	−	−	−	−	−	+	−	+	−	+	ND	*Bacillus megatarium*
21N1, 40N1	+	−	−	−	ND	ND	−	−	−	−	−	ND	*Bacillus* sp.
60X3, 21X1	+	+	−	+	−	−	+	+	−	−	+	+	*B. Citrobacter freundii*
28N2, 60N1	+	+	−	−	ND	ND	−	−	−	+	+	−	*Chryseobacterium* sp.
40E1, 59E2	+		−	−	+	−	+	−		+	−	−	*Enterococcus faecalis*
21E1, 59E3	+	+	−	−	−	−	+	−	−	+	−	+	*Enterobacter aerogens*
59M3, 28E2, 28M1	+	+	+	−	+/−	+	+	+	−	−	−	−	*Escherichia coli*
24N1	+		−	−	+	−	+	−	+	+	+	−	*Enterococcus faecalis*
24M2, 28M2, 28N1, 40M2, 59M2, 60M2	+	−	−	−	−	+	−	−	−	−	−	−	*Morganella* sp.
24E1, 40E2	+	−	−	−	−	−	−	−	−	−	−	−	*Neisseria* sp.
24C1, 59C1, 60C2	+	−	−	−	−	−	+	−	−	−	−	−	*Pseudomonas aeruginosa*
21C1	+	−	−	−	−	+	+	−	−	−	+	+	*Pseudomonas fluorescens*
40X2, 59X1	+	−	−	−	−	−	−	+	−	−	−	−	*Proteus mirabilis*
60X2	+	−	−	−	−	+	−	+	−	−	−	−	*Proteus vulgaris*
28X1	+	−	−	+	−	−	−	+	−	+	−	−	*Proteus penneri*
24X1	+	−	−	−	−	−	+	−	−	+	+	−	*Proteus* sp.
28C3, 40C1	+	−	−	−	−	−	+	−	−	−	−	−	*Pseudomonas* sp.
60X1, 21X2	−	−	−	+	−	−	+	−	−	−	−	−	*Salmonella typhimurium*
24P1	+	+	−	−	−	−	+	−	−	−	−	−	*Staphylococcus aureus*
28P2, 40P1	+	+	−	−	−	−	−	−	+	−	−	ND	*Staphylococcus epidermidis*
28X2, 40X1	−	−	−	+	−	+	+	−	−	−	+	−	*Salmonella paratyphi* A
40M1, 60M1	+	−	+	+	+	−	+	+	−	+	−	+	*Serratia* sp.
21P1	+	+	−	+	−	−	+	−	−	−	−	ND	*Staphylococcus aureus*
59P1, 60P2	+	+	+/−	−	+	−	+/−	−	+	−	−	+	*Staphylococcus* sp.
24N3, 24M1, 28P1, 40T1, 59X2, 59E1, 59M4, 59P2, 60N2, 60C1, 60P1	−/+	−/+	−	−/+	−/+	−	−/+	−	−/+	−	−	−/+	Un identified

a. Glucose; b. Lactose; c. Adonitol; d. Sorbitol; e. Ribose; f. Rhamnose; g. Mannitol; h. Xylose; i. Dextrose; j. Esculin; k. Arabinose; l. Mannose. ND- Not determined

**Fig. 2:**
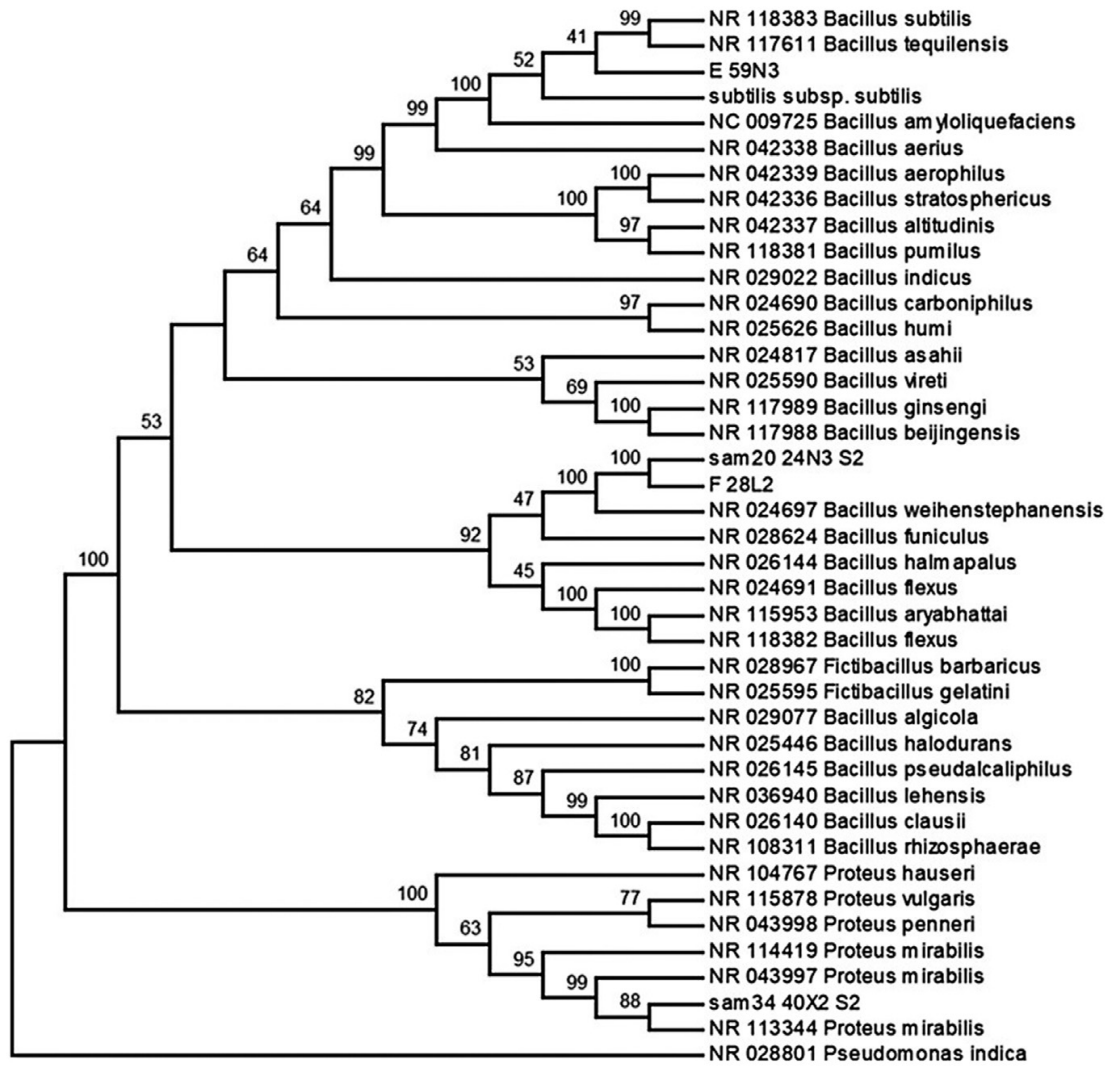
Phylogenetic inference using Neighbor-Joining method in MEGA6. The evolutionary distance was calculated using Kimura 2-parameter method. The sum of branch length of the optimal tree was 0.98420. The bootstrap values were shown above the branches. The analysis involved 41 nucleotide sequences. The bacterial species were isolated from the oral cavity of healthy Indian cobra*, Naja naja.*

Strains such as 24N3, 28L2, 40X2, and 59N3 were further studied for molecular characterization by 16S rRNA sequences. These strains were selected as they showed changeable characters with repeated experiments as well as certain peculiar characteristics. Strain numbers 24N3, 28L2 and 59N3 were identified as *Bacillus* sp. of which 59N3 was further confirmed up to species level by BLAST analysis of the 16S rRNA gene sequence that showed 99% similarity with *Bacillus atrophaeus*. Other two strains, 24N3 and 28L2, were closest to *Bacillus weihenstephanensis* (Fig. 2). Similarly strain number 40X2 is further confirmed up to species level by BLAST analysis of the 16S rRNA gene sequence that showed 99% similarity with *Proteus mirabilis.*

The identified bacteria from oral cavity were classified into four phyla and five classes such as bacilli (firmicutes), γ-proteobacteria and β-proteobacteria (proteobacteria), actinobacteridae (actinobacteria) and flavobacteria (bacteroidetes), represented eleven families- alcaligenaceae, aeromonadaceae, bacillaceae, enterobacteriaceae, enterococcaceae, flavobacteriaceae, micrococcaceae, moraxellaceae, neisseriaceae, pseudomonadaceae and staphylococcaceae (Table 5). Enterobacteriaceae was the leading family followed by pseudomonadaceae and staphylococcaceae, bacillaceae and aeromonadaceae. Other families such as alcaligenaceae, enterococcaceae, flavobacteriaceae, moraxellaceae and neisseriaceae had lower representation (Table 5).

**Table 5:** Summary of incidence of different bacterial species isolated from the oral cavity of healthy Indian cobra.

Phylum	Class	Order	Family	Genus	Species	Number of incidences
Firmicutes	Bacilli	Bacillales	Staphylococcaceae	*Staphylococcus*	*S. aureus* *S. epidermidis*	4 2
		Bacillales	Bacillaceae	*Bacillus*	*B. cereus* *B. megatarium* *B. atrophaeus* *B. weihenstephanensis*	2 2 2 6
		Lactobacillales	Enterococcaceae	*Enterococcus*	*E. faecalis*	3
Proteobacteria	γ- Proteobacteria	Enterobacteriales	Enterobacteriaceae	*Proteus*	*P. mirabilis* *P. vulgaris* *P. penneri*	3 2 2
				*Morganella*	sp.	5
				*Enterobacter*	*E. aerogens*	2
				*Escherichia*	*E. coli*	3
				*Citrobacter*	*C. freundii*	3
				*Serratia*	sp.	2
				*Achromobacter*	sp.	2
				*Salmonella*	*S. typhimurium* *S. paratyphi* A	4 2
		Aeromonadales	Aeromonadaceae	*Aeromonas*	*A. hydrophila* *A. salmonicida*	4 1
		Pseudomonadales	Pseudomonadaceae	*Pseudomonas*	*P. aeruginosa* *P. fluorescens*	3 4
			Moraxella ceae	*Acenetobacter*	*A. baumannii*	3
	β-Proteo bacteria	Neisseriales	Neisseria ceae	*Neisseria*	sp.	2
		Burkholderiales	Alcaligenaceae	*Alcaligenes*	sp.	2
Bacteroidetes	Flavobacteria	Flavobacteriales	Flavobacteriaceae	*Chryseobacterium*	sp.	2
Actinobacteria	Actinobacteridae	Actinomycetales	Micrococcaceae	*Micrococcus*	sp.	1

The oropharynx of the Chinese cobra contained a wide range of bacteria (10 aerobic Gram-positive species, 20 aerobic Gram-negative species and 14 anaerobic species) [6]. Among Gram-negative bacteria, *Morganella morganii* was the commonest pathogen. Other important Gram-negative pathogens included *Aeromonas hydrophila* and *Proteus* species. *Enterococcus faecalis* and coagulase-negative *Staphylococci* were the commonest Gram-positive isolates. Various anaerobic *Clostridium* species were also recorded. Lam et al. [7] studied the oral bacterial flora of the same two species (*N. atra* and *Cryptelytrops albolabris*) from the same locality. Nevertheless, the most common aerobic Gram-positive bacteria were *Enterococcus faecalis, Tsukamurella* species and coagulase-negative *Staphylococcus*. A total of 41 aerobic Gram-negative bacteria species were cultured from these two species of snakes, with *Morganella morganii, Pseudomonas aeruginosa* and *Stenotrophomonas maltophilia* being the most common. Among anaerobic bacteria, the most common isolates were *Clostridium bifermentans, Clostridium baratii/sardiniense* and *Clostridium perfringens.* Recently, Shaikh et al. [8] studied cultivable oral bacterial flora of important venomous snakes of India where Indian cobra was included (N = 5). These authors reported 27 aerobic Gram-positive and 60 aerobic Gram-negative bacteria mostly dominated by enterobacteriaceae. Results of the present study matches well with Shaikh et al. [8].

Chinese cobra harbored more bacteria in the oral cavity compared to both venomous (*C. albolabris*) and non-venomous snakes in terms of total number of species, both pathological and non-pathological [7]. The diversity of the oral microbiota of the snakes can be considered as nonspecific and associated with the environment, animal feeding habits and seasonality [9, 10]. *Clostridium* was the dominant genera in the Chinese cobra. However, *Clostridium* species were not recorded during the present study as we did not perform anaerobic cultures. The genus *Neisseria* is a Gram-negative coccus which mainly includes non-pathogenic species such as *N. sicca* and *N. flavescens*, These are the common members of the oral bacterial community of humans [11]. The same could not be identified in the present study, due to their complex biochemical and physiological properties. Moreover, *Acinetobacter baumannii****,*** *Aeromonas hydophilla****,*** *Citrobacter diversus****,*** *C. freundii****,*** *Enterococcus faecalis, Enterobacter aerogens****,*** *Escherichia coli, Morganella* sp., *Proteus mirabilis****,*** *P. vulgaris****,*** *Pseudomonas aeruginosa, Serratia* sp. and *Staphylococcus aureus* were some of the important pathogens identified in the present study and these bacteria had also been recovered from cobra bite wounds [10, 12].

**Fig. 3:**
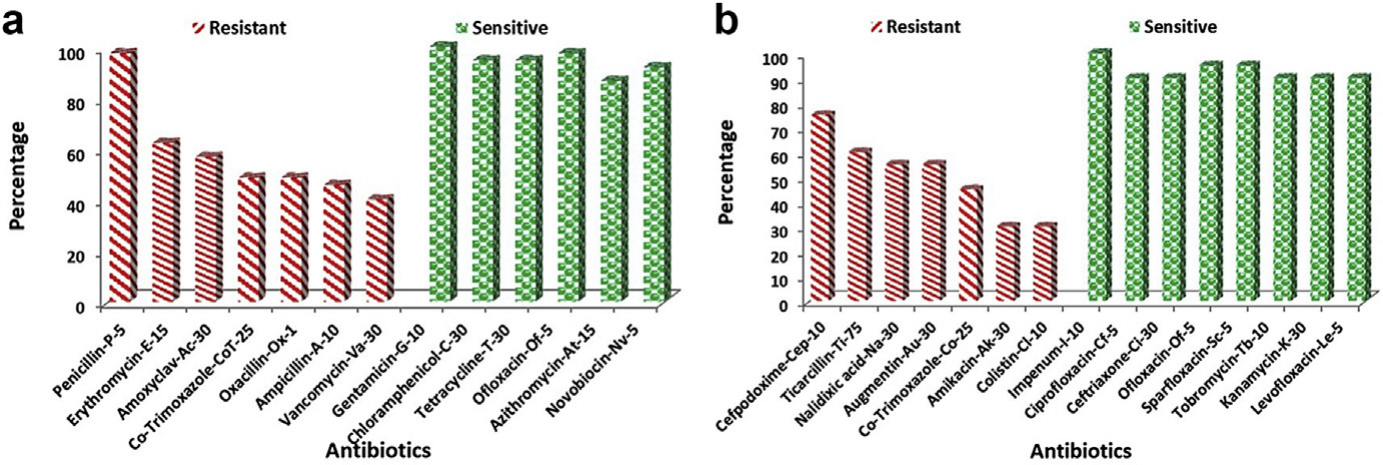
(a)- Comparison of percentage of occurrence of resistant and sensitive antibiotics among isolates from oral cavity of *Naja naja* using ICOSA 20-plus. ((b)- Comparison of percentage of occurrence of resistant and sensitive antibiotics among isolates from oral cavity of *Naja naja* using ICOSA 20-minus.

Most of the isolates were resistant to antibiotics like Penicillin (97%), Cefpodoxime (75%), Ticarcillin (60%), Erythromycin (62%), Amoxyclav (57%), Nalidixic acid (55%), Augmentin (55%) and Co-Trimoxazole (49%). However, the isolates were sensitive to antibiotics viz. Imipenem, Ciprofloxacin, Ceftriaxone, Chloramphenicol, Tetracycline, Ofloxacin, Gentamicin, Sparfloxacin, Tobromycin and Novobiocin (≥90%) (Fig. 3 a & b).

In India, doctors usually prescribe broad spectrum antibiotics which results a low incidence of wound infection after cobra bites. However, use of antibiotics in the management of snakebite has been criticized by many researchers [13, 14]. In the present study, the antibiograms of isolated strains revealed the presence of antibiotic resistant pathogens in the oral cavity of snakes. Since similar types of bacteria were also recorded from the mouth swabs of cobra from diverse localities and snake bite wounds, it is necessary to administer proper antibiotics. Shaikh et al. [8] through a similar study from Maharastra (India) suggested antibiotics like Azithromycin or Amoxicillin/Clavulanic acid for Gram-positive and Imipenem or Levofloxacin for Gram-negative microorganisms. We also do not propose prescription of resistant antibiotics such as Amoxyclav, Ampicillin, Oxacillin, Penicillin and Cefpodoxime. However, these broad spectrum antibiotics are in normal practice in India and elsewhere like Ampicillin in Saudi Arabia and Eastern Ecuador [15]; Benzylpenicillin in Zimbabwe [16]; Ampicillin plus Cloxacillin and Ampicillin alone or Benzylpenicillin in Hong Kong [17, 18] and Amoxicillin/Clavulanate in Chinese cobra bite [7]. In case of severity with established infections, the best way is to culture the isolates and screening for different antibiotics before beginning of the treatment.

## Declarations

### Author contribution statement

Sujogya Kumar Panda: Conceived and designed the experiments; Performed the experiments; Analyzed and interpreted the data; Contributed reagents, materials, analysis tools or data; Wrote the paper.

Laxmipriya Padhia: Conceived and designed the experiments; Performed the experiments.

Gunanidhi Sahoob: Conceived and designed the experiments; Analyzed and interpreted the data, Wrote the paper.

### Funding statement

Sujogya Kumar Panda was supported by SERB, Govt. of India (SB/FT/LS-252/2012), Laxmipriya Padhi and Gunanidhi Sahoo are supported by the UGC-SAP programme and UGC MRP (37-282/2009-SR), Govt. of India for financial support.

### Competing interest statement

The authors declare no conflict of interest.

### Additional information

Data associated with this study is available at NCBI (National Center for Biotechnology Information) under the accession number KX164444, KX495210, MF084216 and MF084215.
